# A281 GASTROPARESIS AND FUNCTIONAL GASTROPARESIS IN YOUNG CANADIAN WOMEN: A DETAILED PRACTICE REVIEW IN LONDON, ONTARIO

**DOI:** 10.1093/jcag/gwac036.281

**Published:** 2023-03-07

**Authors:** Y Han, K S McIntosh, A N Rahman

**Affiliations:** Western University, London, Canada

## Abstract

**Background:**

Gastroparesis is a chronic clinical syndrome characterized by delayed gastric emptying in the absence of mechanical obstruction causing symptoms such as nausea, vomiting, and abdominal pain. This condition is heterogenous in its severity and the resulting impact on individuals who experience this diagnosis. Patients with severe gastroparesis symptoms can require extensive pharmacologic and nutritional therapies.

**Purpose:**

We hope to identify young women (age<50) experiencing gastroparesis in local gastroenterology clinics to describe a group of patients requiring extensive therapeutic support suggestive of a severe subset of this condition.

**Method:**

Mutual patients with a diagnosis of gastroparesis between a London motility clinic and nutrition clinic were identified. Key patient characteristics including current age, age at diagnosis, current BMI, gastric emptying scan results, the number of prokinetic/anti-constipation/anti-emetic medications ever used, the presence of opioid use, the use of outpatient IV access/IV fluids, the placement of a feeding tube, and the use of total parental nutrition were recorded through a retrospective chart review. Descriptive analyses were performed on the recorded data.

**Result(s):**

A total of 26 patient charts were reviewed and 11 patients meeting inclusion criteria were included in the analysis. The mean age was 32.7y, the mean age at gastroparesis diagnosis was 28.5y, and the mean body mass index was 22.9kg/m^2^. Eighty-two percent of these patients have had a gastric emptying scan to confirm their diagnosis, and 36% of them have experienced multiple scans. The mean gastric emptying half-time was 231 minutes and the mean retained gastric content at 4 hours post-ingestion was 39.5%. The mean number of motility medications ever used was 2.2, the mean number of anti-constipation medications ever used was 2.1, and the mean number of anti-emetic medications ever used was 1.5. Fifty-five percent were prescribed opioid medications. The majority of patients identified required advanced nutritional strategies including 45% requiring outpatient IV access and IV fluid hydration, 55% ever using TPN, and 73% ever using a feeding tube.

**Conclusion(s):**

We are encountering an increasing number of gastroparesis patients who require intensive therapy and resource allocation. These patients outline the need for further health resources and integration of care from practitioners across multiple disciplines for optimal management.

**Please acknowledge all funding agencies by checking the applicable boxes below:**

None

**Disclosure of Interest:**

None Declared

